# A novel case of lupus nephritis and mixed connective tissue disorder in a COVID-19 patient

**DOI:** 10.1016/j.amsu.2022.103653

**Published:** 2022-04-23

**Authors:** Sajjad Ali, Talal Almas, Ujala Zaidi, Farea Ahmed, Sufyan Shaikh, Fathema Shaikh, Rida Tafveez, Maaz Arsalan, Ishan Antony, Meetty Antony, Burhanuddin Tahir, Abdullahi T. Aborode, Murtaza Ali, Vikneswaran Raj Nagarajan, Arjun Samy, Maen Monketh Alrawashdeh, Maha Alkhattab, Joshua Ramjohn, Jeremy Ramjohn, Helen Huang, Qassim Shah Nawaz, Kashif Ahmad Khan, Shane Khullar

**Affiliations:** a Department of Medicine, Ziauddin Medical University, Karachi, Pakistan; b RCSI University of Medicine and Health Sciences, Dublin, Ireland; c Department of Medicine, Karachi Medical and Dental College, Karachi, Pakistan; d Department of Medicine, Dow University of Health Sciences, Karachi, Pakistan; e University of Toronto, Toronto, Canada; f Jawaharlal Nehru Medical College, Belgaum, India; g Healthy Africans Platform, Research and Development, Ibadan, Nigeria; h Department of Medicine, Dr. Ruth K.M. Pfau, Civil Hospital Karachi, Pakistan; i Department of Surgery, Galway University Hospital, Galway, Ireland; j Sligo University Hospital, Sligo, Ireland; k University of Birmingham Medical School, Birmingham, UK; l National University of Ireland Galway, Galway, Ireland

## Abstract

**Introduction:**

Mixed connective tissue disease (MCTD) is a rare autoimmune condition characterized by Scleroderma, Polymyositis, and Systemic Lupus Erythematous (SLE). Though a possible relationship between COVID-19 and autoimmune diseases has been recently reported, its pathophysiological mechanism behind flares in Lupus Nephritis (LN), a complication of SLE, remains unknown.

**Case presentation:**

A 22-year-old COVID-19 positive female presented with anemia, bilateral pitting edema, periorbital swelling, and posterior cervical lymphadenitis. Further inspection revealed lower abdominal striae, hepatosplenomegaly, and hyperpigmented skin nodules. Complete blood counts showed elevated inflammatory markers and excessively high protein creatinine ratio. Antinuclear antibody titers were elevated (anti-smith and U1 small nuclear ribonucleoprotein) and Rheumatoid Factor was positive. She was diagnosed with MCTD associated with a flare of LN. To control her lupus flare, a lower dose of steroids was initially administered, in addition to oral hydroxychloroquine and intravenous cyclophosphamide. Her condition steadily improved and was discharged on oral steroid maintenance medication.

**Discussion:**

We present a rare phenomenon of newly diagnosed LN, a complication of SLE, with MCTD in a PCR-confirmed COVID-19 patient. The diagnostic conundrum and treatment hurdles should be carefully addressed when patients present with lupus and COVID-19 pneumonia, with further exploration of the immuno-pathophysiology of COVID-19 infection in multi-systemic organ dysfunction in autoimmune disorders.

**Conclusion:**

In COVID-19 patients with LN and acute renal injury, it is critical to promptly and cautiously treat symptomatic flares associated with autoimmune disorders such as SLE and MCTD that may have gone unnoticed to prevent morbidity from an additional respiratory infection.

## Highlights

• SLE disease has been associated with COVID-19. However, there is a lack of data on LN in conjunction with MCTD in COVID-19 positive patients.
• A possible relationship between Coronavirus disease 2019 (COVID-19) and autoimmune disease has been documented in many case reports.
• Because of the overlapping clinical manifestations and laboratory findings between lupus and COVID-19 pneumonia, the diagnostic problems and treatment hurdles should be carefully addressed.
• In COVID-19 patients with LN flare and acute renal injury, it is critical to resolve any reversible causes of the kidney injury and manage the COVID-19 before treating the LN.

## 1 Introduction

Mixed Connective Tissue Disease (MCTD) was initially identified in 1972 as a condition characterized by overlapping characteristics of systemic sclerosis, systemic lupus erythematosus (SLE), and polymyositis [1]. Because the signs and symptoms of these three diseases may not always emerge simultaneously, diagnosing MCTD can be difficult. SLE is a chronic inflammatory autoimmune disease that presents a wide range of clinical symptoms owing to its influence on several organ systems, with Lupus Nephritis (LN) being one of the disease manifestations. LN affects up to five out of ten people with SLE and can manifest clinically as weight gain, hypertension, and foamy urine [2]. Despite emerging developments in the treatment of Lupus Nephritis, guidelines for management are not definitive and only consist of symptomatic relief globally [3].

Severe Acute Respiratory Syndrome Coronavirus 2 (SARS-CoV-2), which causes Coronavirus Disease-2019 (COVID-19), has been a global epidemic for the past two years. COVID-19 causes a broad spectrum of clinical symptoms that impact various body systems, often appearing with respiratory signs and symptoms, such as flu-like illness exacerbated by acute respiratory distress syndrome (ARDS) and lung failure [4,5]. Additional symptoms and risks include severe metabolic syndrome, acute renal injury, neurological diseases, cardiovascular and thromboembolic events such as encephalopathy, seizures, and stroke [[6], [7], [8], [9], [10]]. A possible relationship between COVID-19 and autoimmune diseases such as SLE has also been recently documented in many case reports within the literature [[11], [12], [13]]. However, there is a lack of data and knowledge on LN in conjunction with MCTD in COVID-19 positive patients. Given the clinical importance of COVID-19 during the ongoing pandemic, the present paper elucidates a rare case of newly diagnosed LN in combination with MCTD in a PCR-confirmed COVID-19 patient. A review of the literature was conducted to analyse all linked clinical case reports and case series to provide an in-depth understanding of the relationship between COVID-19 and renal manifestations of Lupus.

## 2 Case Presentation

A 22-year-old COVID-19 positive female presented to the emergency department via an ambulance with fever, weight loss (20 kg), shortness of breath, loose stools, and multiple skin lesions present for the previous eight months. The fever was mild, intermittent, and alleviated by antipyretics. This was accompanied by frequent bowel movements (4–5 times per day) and progressive shortness of breath at rest and during exertion. However, there were no reports of orthopnea or paroxysmal nocturnal dyspnea. She also complained of polyuria and hematuria for the last two days. Her past medical history was insignificant, and other aspects of her health, including menstrual health, were unremarkable. There is no history of chronic disease in her family.

**Fig. 1:**
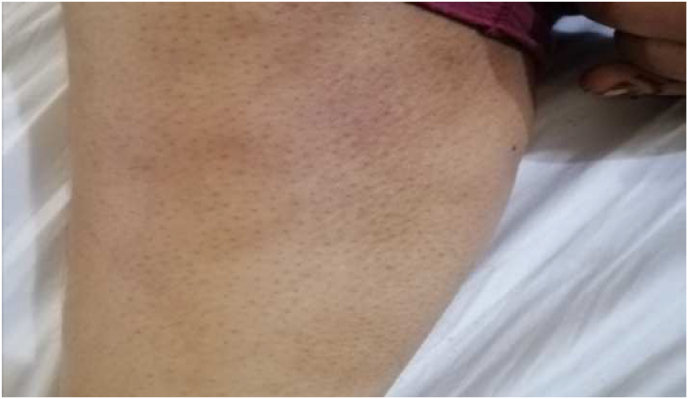
Hyperpigmented lesion on the leg.

Upon presentation to the emergency department, she was a-febrile, tachypneic but hemodynamically stable, and well oriented to time and place. On inspection, there was a noticeable pallor, indicating a positive anemic state. Dehydration, bilateral pitting edema up to the shin, and periorbital swelling were also seen. Posterior cervical lymph nodes (less than 0.4cm) and a lymph node (1 cm) in the right axilla were palpable. Painful, itchy, indurated, and hyperpigmented lesions [Fig. 1] were observed in various places of her body, as well as a history of hair loss, mouth ulcers, and mouth dryness.

Abdominal examinations revealed striae over the lower abdomen, a palpable spleen, and a liver with a 17-cm span. Furthermore, several cystic lesions were noticeable on breast examination, with the largest one measuring 1.4 × 0.9 cm in the right breast and 1.4 × 0.6 cm in the left breast. The lesions had a firmness and smooth edges.

Extensive investigations were carried out to rule out any potential diagnoses [Table 1]. A Complete Blood Count (CBC) profile revealed that the patient had low hemoglobin levels. An in-depth analysis of anemia resulted in the reporting of an increased reticulocyte count. Other cell lines were also deranged, with a high leukocyte count and thrombocytopenia. Based on the findings, further screening of inflammatory markers, such as erythrocyte sedimentation rate (ESR) and C-reactive protein (CRP), revealed unusually increased serum levels for both.

**Table 1:** Baseline Laboratory investigations.

Test Name	Results	Normal Ranges
Complete blood count [CBC]		
Hemoglobin	6.3g/dl	12–16 g/dl
HCT	20.10	0.37–0.47
MCV	82 fl	80-100 fl
MCH	25.7 pg	21–32 pg
MCHC	31.3 Gm/dl	33.4–35.5 Gm/dl
TLC	17.5/uL	3.6–110/uL
Neutrophils	73%	55–70%
Lymphocytes	21%	20–40%
Monocytes	4%	2–8%
Eosinophils	2%	1–4%
PLT	660 × 10^6 mcL	150–450 × 10^9 mcL
Reticulocyte Count	1.3%	0.2–2%
Inflammatory Markers		
CRP	153 mg/L	<5 mg/L
ESR	102 mm/hr	3–9 mm/hr
Fasting Lipid Profile		
Cholesterol	240 mg/dl	<200 mg/dl
Triglycerides	612 mg/dl	35–135 mg/dl
LDL	140 mg/dl	<130 mg/dl
Total Lipid	1202 mg/dl	<150 mg/dl
HDL	26 mg/dl	<50 mg/dl
Protein creatinine ratio	9.3g/day	<0.2/day
Urine Direct Report		
Quantitiy	40 ml	800–2000 ml
Colour	Dark Yellow	Pale Yellow
Ph	6.0	4.5–8
Specific Gravity	1.020	1.005–1.025
Albumin	+++	<30 mg/g
Sugars	Nil	0–0.8 mmol/L
Blood (RBCs)	++	≤3
Red Cells (per hpf)	12–13	≤2
Pus cells	2–4	0–4
Nitrites	Nil	Nil
Granular Cast	++	Nil
Amorphous urate	++	–
Miscellaneous Tests		
Total Protein	7.6 g/dL	6–8.3 g/dL
Serum Albumin	1.3 g/dL	3.4–5.4 g/dL
Serum Globulin	6.3 g/dL	2–3.5 g/dL
Albumin/Globulin ratio	0.21	1.1–2.5
D dimer	0.2	<0.5
Lactose Dehydrogenase (LDH)	514 U/L	140–280 U/L

Furthermore, the urine report was positive for protein, red blood cells, pus cells, granular casts, and urate but harmful for any infectious organism. The protein creatinine ratio was excessively high, indicating severe proteinuria. A comprehensive stool culture was also performed, which revealed the presence of mucus, red blood cells, pus cells, and yeast cells. Further investigation included viral markers, which were shown to be negative.

On suspicion of autoimmune disorders [Table 2], antinuclear antibodies (ANA) titers were carried which were found to be elevated. Along with this, Extractable Nuclear Antigen (ENA) profile revealed an increase in antibody titers to the anti-smith (Sm) and U1 small nuclear ribonucleoprotein (U1-RNP). Moreover, she was tested positive for Rheumatoid factor, while C3 and C4 complement levels were within range (see Table 3).

**Table 2:** ANA-ENA Profile testing.

Test Name	Results
ANA (Anti-nuclear antibodies)	Positive
ASMA	Negative
AMA	Negative
Serum Anti-dsDNA (IgG)	Negative
Rheumatoid Factor	Negative
Serum C3	1.21
Serum C4	0.35
Extractable Nuclear Antigen (ENA) PROFILE	
U1-RNP-Antibodies	43.49 U/ml
SS-A/Ro- Antibodies	0.54 U/ml
SS-B/La- Antibodies	0.66 U/ml
Sm-Antibodies	>40 U/ml
Scl-70 Antibodies	1.93 U/ml

**Table 3:** Summary of all the case reports and case series related to lupus in association with COVID-19.

	Authors	Country	Age, Gender	Disease duration	Lupus system involvement	Lupus medications	Severity of COVID-19
14	Watchmake J.M. et al.	United States	60 years, F	33 days	Respiratory, neurological	Steroids, rituximab, methotrexate, remdesivir, apixaban	Mild
15	Kreuter, A. et al.	Germany	79 years, M	NA	Cutaneous, Musculoskeletal	hydroxychloroquine 200 mg twice daily and tapered intravenous glucocorticosteroid therapy	Not infected but vaccinated
16	Brockman, T. et al.	United States	71 years, F	90 days	Renal, respiratory, cardiac	Initially, Clopidogrel and heparin (discountinued later) followed by aspirin and colchicine	Severe
17	Muyldermans, A. et al.	Belgium	56 years, M	127 days	Respiratory, gastrointestinal	hydroxychloroquine 200 mg twice a day	Moderate
18	Roncati, L. et al.	Italy	44 years, M	8 days	Respiratory, neurologic	N.A	Moderate
19	Patil, S. et al.	India	22 years, F	N.A	Musculoskeletal, cutaneous	prednisolone (50 mg daily) (tapered later) hydroxychloroquine (400 mg daily), mycophenolate mofetil (2 g daily), furosemide (20 mg daily), telmisartan (20 mg daily), folic acid, calcium, and vitamin D3	(SLE) following COVID-19 vaccination with Covishield
20	Nespola, M. et al.	Italy	47 years, F	25 days	Vascular	low-dose oral corticosteroids	Severe
21	Karsulovic, C. et al.	Chile	28 years, M	3 weeks	Respiratory, cutaneous	Hydroxychloroquine, Mycophenolate Mofetil 2 g a day Prednisone 20 mg a day with descending tapering	Mild
	Karsulovic, C. et al.	Chile	25 years, F	4 weeks	Articular, hematologic and cutaneous	Hydroxychloroquine, Mycophenolate Mofetil 1 g a day (reinitiated) Prednisone 40 mg a day with descending tapering	Mild
	Karsulovic, C. et al.	Chile	68 years, F	4 weeks	Articular and cutaneous	Hydroxychloroquine, Prednisone 20 mg a day with descending tapering	Mild
22	Yusuf, A.S. et al.	Malaysia	30 years, F	2 weeks	Renal, respiratory, cutaneous	Methylprednisolone 50mg daily) and oral hydroxychloroquine 200mg once daily	Mild
23	Hali, F. et al.	Morocco	25 years, F	19 days	Cutaneous, musculoskeletal, ophthalmic, cardiovascular and hematological	Methylprednisolone	Mild
24	El Aoud, S. et al.	France	62 years, M	39 days	Respiratory, renal, musculoskeletal, neurologic	methylprednisolone 120 mg IV for 2 repeated doses, tocilizumab (TCZ) at 600 mg, and Tazocilline. Two days later, corticoids were decreased to 80 mg for 2 days then 40 mg for 2 more days	Severe
25	Bahramnezhad, F. et al.	Iran	56 years, M	N.A	Vascular	dexamethasone 8 mg three times daily (intravascular), hydroxychloroquine tablets 200 mg twice daily, remdesivir injection 200 mg on day 1 and 100 mg from day 2 to day 5, and interferon-beta 250 mg every 48 hours (subcutaneous)	Mild
26	Kincaid, K.J. et al.	United States	43 F	N.A	Hematological, Neurological	mycophenolate and hydroxychloroquine	Mild
27	Smeele, H.T et al.	Netherlands	31 years, F Gravida 1, para 0, gestational age of 38 weeks F	N.A	Musculoskeletal	azathioprine (25 mg/day), hydroxychloroquine (200 mg/day), prednisone (5 mg/day). Prophylactic acetyl sialic acid was initiated after pregnancy was confirmed	Mild
	Smeele, H.T et al.	Netherlands	39 years, F	N.A	Musculoskeletal, renal	Hydroxychloroquine, azathioprine and etanercept. Prophylactic acetyl sialic acid was initiated after pregnancy was confirmed.	Mild
28	Gracia-Ramos, A.E. et al.	Mexico	45 years, M	N.A	Hematological, Musculoskeletal, Respiratory	Pulse methylprednisolone therapy (1 g IV for 5 days) and chloroquine 150 mg per day	Moderate
29	Plotz, B. et al.	United States	27 years, F	N.A	Cutaneous, gastrointestinal, Vascular	Enoxaparin, Apixaban	Mild
30	Zamani, B. et al.	Iran	39 years, M	6 weeks	Cutaneous, renal and neurological	Pulse methylprednisolone (1000 mg for three consecutive days) continued with hydroxychloroquine and prednisolone	Mild
31	Domínguez-Rojas, J. et al.	Peru	11 years, M	N.A	Musculoskeletal, gastrointestinal, cutaneous	IV immunoglobulin, acetylsalicylic acid and methylprednisolone acetate. Post biopsy: chemotherapy including etoposide, cyclosporine, dexamethasone, and methotrexate	Moderate
32	Cohen, M.K. et al.	Israel	62 years, F	2 months	Gastrointestinal, renal	low-dose prednisone, hydroxychloroquine, eltroxin, pregabalin, rosuvastatin, carbamazepine, ramipril, and clopidogrel	Mild
33	Pang, J.H.Q. et al.	Singapore	30 years, M	7 days	Gastrointestinal, Vascular	low-molecular-weight heparin at 1 mg/kg, enoxaparin sodium injections	Mild
34	Ghafouri, S. et al.	United States	89 years, M	N.A	Musculoskeletal	Patient non-compliant with medications	Critical
35	Shoskes, A. et al.	United States	69 years, M	N.A	Cutaneous, renal and neurological	N.A	Mild
36	Guven, F. et al.	Turkey	43 years, F	N.A	Neurological, hematological	N.A	Mild
37	Araten, D.J. et al.	United States	39 years, F	9 days	Vascular	eculizumab since the age of 28	Mild
	Araten, D.J. et al.	United States	54 years, F	3 months	Gastrointestinal, Vascular, Hematological	Eculizumab, tacrolimus, mycophenolate, low doses of prednisone, and hydroxychloroquine	Mild
	Araten, D.J. et al.	United States	60 years, F	N.A	Vascular	Eculizumab	Mild
38	Bonometti, R. et al.	Italy	85 years, F	N.A	Hematological, Renal, Neurological	hydroxychloroquine	Moderate
39	He, F. et al.	China	39 years, F	32 days	Hematological, Renal, Musculoskeletal	Prednisone, hydroxychloroquine, mycophenolate mofetil	Severe
40	Cardoso, E.M. et al.	United States	18 years, F	17 days	Renal, Hematological	ceftazidime, vancomycin, azithromycin, and hydroxychloroquine	Severe
41	Gemcioglu, E. et al.	Turkey	34 years, F	N.A	Neurological	acetyl salicylic acid, enoxaparin, favipiravir, hydroxychloroquine and azithromycin	Moderate
42	Yarlagadda, K. et al.	United States	31 years, M	N.A	Respiratory, Hematological	N.A	Moderate
43	Cho, J. et al.	Japan	58 years, F	N.A	Hematological	prednisolone	Asymptomatic
	Cho, J. et al.	Philippines	32 years, F	N.A	Renal	hydroxychloroquine, mycophenolate mofetil and prednisolone	Moderate
	Cho, J. et al.	Philippines	29 years, F	N.A	Renal	hydroxychloroquine, azathioprine and low-dose prednisolone	Moderate
44	Arpali, E. et al.	Turkey	28 years, F	N.A	Renal	Cyclophosphamide 500 mg/m2/mo for 7 months, mycophenolate mofetil, oral corticosteroids	Mild
45	Grimminck, K. et al.	Netherlands	31- years, F G1P0, 38 + 1 weeks pregnant	N.A	N.A	Methyldopa, prednisolone and azathioprine	Mild
46	Kichloo, A. et al.	United States	22 years, F	5 days	Respiratory, Renal and Cardiac	Hydroxychloroquine, mycophenolic acid	Moderate

Legends: N.A: Not Available, M: Male, F: Female, mg: milligram.

Ultrasonography was performed to thoroughly assess breast tissue, which revealed several cystic regions in the right breast, primarily in the upper quadrant. One measured 16.2 × 9.4 mm and extended into the retro-areolar area, displaying diffuse internal echoes. Multiple large lymph nodes measuring 16.0 × 9.4 mm were seen in the right axilla, along with hilar thinning. Multiple cystic regions were found dispersed throughout the parenchyma of the left breast, one of them being next to the areolar edge and measuring 15.8 × 6.8 mm. The discovered cysts were most likely complicated cysts. The left axilla showed a few swollen lymph nodes measuring 22.0 × 10.0 mm, as well as thinning of the hilum.

Echocardiography was performed to rule out cardiac involvement, which was expected. Along with an endoscopy, a color Doppler of the lower limbs was performed. Endoscopy revealed minor pangastritis, and a biopsy was performed (results are awaited). A Doppler examination of the lower limbs revealed no indications of stenosis, occlusion, or thrombosis. However, it did indicate bilateral soft tissue edema and a benign-looking inguinal lymph node on the right side.

The on-call nephrologist ordered a renal biopsy for further confirmation, and the results are still pending. Based on the clinical findings and laboratory investigations, the patient was diagnosed with MCTD associated with a flare of LN.

Despite the initial concerns regarding the commencement of steroids in an active COVID-19 infection, the management team decided to control her lupus flare with a lower steroid dose (intravenous methylprednisolone 50mg once daily) throughout hospitalization, in addition to oral hydroxychloroquine 200mg once daily. The patient was also given 1g intravenous cyclophosphamide once a month. Her condition steadily improved, and she was stable on the 7th day of her hospitalization. She was discharged on oral steroid maintenance medication with a follow-up appointment. At the follow-up appointment, the patient continues to do well with no evidence of recent flare-up and a complete resolution of her acute symptoms.

The present paper has been reported in accordance with the SCARE guidelines [14].

## 3 Methods

We conducted a thorough review of the literature and collated all clinical cases of LN and/or MCTD linked with COVID-19 infection, taking into account their place of origin, age, sex, body systems involving the disease, its associated medical regimen, and the severity of COVID-19 condition. We conducted a literature search on Pubmed using the terms ‘lupus nephritis', ‘systemic lupus erythematosus, ‘SLE,’ ‘Mixed Connective Tissue Disease,’ ‘MCTD,’ ‘COVID-19′, and ‘SARS-CoV-2'. The study included all case reports and case series. Articles that lacked extractable clinical data and a description of individual data were eliminated. The titles and abstracts of the retrieved publications were used to determine their eligibility. The eligibility criteria were met by a total of 33 papers involving 37 patients (Table 2).

## 4 Results

Out of the total papers, eleven articles were from Asia [19,22,25,30,32,33,36,39,41,43,44], eight from Europe [15,17,18,20,24,27,38,45], eleven from North America [14,16,26,28,29,34,35,37,40,42,46], two from South America [21,31] and one from Africa [23].

These Lupus patients were predominantly female (female/male ratio: 27:10). Fourteen of the cases had underlying LN. At the same time, there was only one patient who had underlying MCTD [21]. Moreover, most of the cases had musculoskeletal involvement [15,19,23,24,27,28,31,34,39].

For lupus management, more than half (56.7%) of the patients were on hydroxychloroquine therapy. Moreover, about half of the patients were given corticosteroids, while only nine were on mycophenolate mofetil.

We have analyzed and classified COVID-19 based on its severity, including asymptomatic, mild, moderate, severe, or critical. The majority of the patients (83.7%) were infected with mild to moderate COVID-19. In contrast, seven (18.9%) of the patients had severe to serious COVID-19. Except for 14 individuals, everyone was given systemic steroid therapy. Eculizumab was administered to three of the patients [37]. Tocilizumab IV was administered to a single patient [24]. Furthermore, for acute renal injury, only one patient required hemodialysis [40]. COVID-19 was linked to seven cases of thromboembolic events [20,25,29,33,37,41].

The clinical symptoms of active SLE and COVID-19 infection are often overlapping. Fever, rash, arthralgia, malaise, acute renal damage, and cytopenias are also symptoms of both disorders. Only four instances were documented to have a flare of lupus during the COVID-19 infection, according to our research [21,22,27,46].

## 5 Discussion

The relation of acute exacerbations of rheumatic and connective tissue diseases with viral infections like HIV, poliomyelitis, and influenza [47,48]. Because of the current COVID-19 pandemic, attention has been drawn to the possible flare-ups seen in patients with SLE and MCTD associated with mild COVID-19 infection, including diffuse lymphadenopathy [21] and full-blown SLE vasculitis [38]. A study by Jose L Pablos et al. statistically demonstrated how severe COVID-19 infection was a risk factor in diagnosing connective tissue disease, omitting inflammatory arthritis [49]. Moreover, Cheng Chen et al. reported in their study that during the COVID-19 pandemic, patients diagnosed with SLE abruptly ceased taking immunosuppressive therapy, which led to rapid flare-ups in their autoimmune conditions [50].

In this case, the patient had SLE and MCTD symptoms that were not recognized until she experienced a suspected flare-up of LN. During her active course of COVID-19 infection, she developed new-onset hematuria, proteinuria, bilateral pitting pedal edema, and periorbital edema, all of which were suggestive of Lupus Nephritis flare-up. Our patient was tested for autoimmune serology and found to have elevated levels of Anti-SM Antibodies, as well as ANA and Anti-U1 RNP Antibodies. Certain clinical features that confirm the diagnosis of SLE with MCTD include posterior cervical lymphadenitis, rheumatoid skin nodules, elevated inflammatory markers (ESR, CRP), and a deranged cell lineage. Though our patient was not commenced on immunosuppressive therapy during her illness, it did not affect her normal daily activities. This created the notion that COVID-19 infection may be associated with flare-ups in autoimmune disorders such as SLE and MCTD, which has not previously been documented in the literature.

The literature search primarily yielded case reports and case series involving the aforesaid patient population. The cohort size in the included studies was mainly limited to individual cases given the dearth of data and evolving COVID-19 literature. Furthermore, the follow-up duration for all of the studies was noted to be homogenous. The ongoing debate regarding the plight of SLE diagnosed individuals for an increased risk of acquiring COVID-19 infection due to immune dysregulation has already been assessed in a study of more than 900 patients (91% females) with the negative outcome of this hypothesis in which SLE diagnosed patients taking immunosuppressant like hydroxychloroquine and mycophenolate mofetil were not found to have an increase in COVID-19 infectivity rate [51]. Similarly, another study showed the same results, stating that patients with Lupus and the general population share the same COVID-19 hospitalization risk factors [52]. However, Giuseppe A. Ramirez et al. [48] concluded that COVID-19 could have a moderately increased morbidity in patients suffering from SLE, even though the study had certain limitations and selection bias, rendering the possibility controversial. In addition, another complication arising from overlap in symptoms of rheumatic flare and COVID-19 was observed in a retrospective study conducted in Tongji hospital, which stated that the overlapped symptoms were a cause of increased morbidity due to delayed diagnosis in patients presenting with respiratory infection due to COVID-19 [53]. Our patient, who presented with COVID-19 results but was later identified with chronic MCTD associated with LN, was a case that was somewhat but not entirely similar.

The onset of post-COVID-19 vaccine-associated SLE has also been reported in a case study by Miranda et al. [54], supporting the fact that COVID-19, as a multisystemic infection, has possible immune dysregulation mechanisms and antigen-autoantibody interactions, supporting the evidence of new-onset kidney disease in genetically susceptible individuals such as our patient. Our case is the first in our region to describe a newly diagnosed nephritic illness coupled with SLE and MCTD in a PCR-confirmed COVID-19 infected woman. The rarity of this occurrence suggests that it should be included in the literature.

## 6 Conclusion

We presented a case report of a PCR-confirmed COVID-19 positive patient with LN in association with SLE and MCTD. Because of the overlapping clinical manifestations and laboratory findings between lupus and COVID-19 pneumonia, the diagnostic problems and treatment hurdles should be carefully addressed. In COVID-19 patients with LN and acute renal injury, it is critical to promptly treat symptomatic flares associated with autoimmune disorders such as SLE and MCTD that may have gone unnoticed to prevent morbidity from the addition of a respiratory infection. However, the commencement of steroids at lower doses to treat lupus flare should be considered with caution in an active COVID-19 infection. To validate or reject the current findings, more extensive prospective studies are needed.

## Ethical approval

NA.

## Sources of funding

N/A.

## Author contribution

SA, TA, UZ, FA, SS, FS: conceived the idea, designed the study, and drafted the manuscript, RT, MA, IA, MA, BS, ATA: Curated the literature review table and revised the first draft of the paper critically, MA, VRN, AS, MMA, MA: conducted literature search and screened the studies to fit the inclusion and exclusion criteria for the paper, JR, JR, HH: revised the manuscript critically and refined the literature review table based on reviewer comments, QSN, KAK, SK, SA, TA: revised the final version of the manuscript critically and gave the final approval.<a name = "Line_manuscript_48">

## Registration of research studies

Name of the registry: NA.

Unique Identifying number or registration ID: NA.

Hyperlink to your specific registration (must be publicly accessible and will be checked): NA.

## Guarantor

Talal Almas, RCSI University of Medicine and Health Sciences, 123 St. Stephen's Green, Dublin 2, Ireland, Talalalmas.almas@gmail.com.

## Consent

Written informed consent was obtained from the patient for publication of this case report and accompanying images. A copy of the written consent is available for review by the Editor-in-Chief of this journal on request.

## Provenance and peer-review

Not commissioned, externally peer-reviewed.

## Declaration of competing interest

N/A.
